# Hypoglycemia during hyperosmolar hyperglycemic crises is associated with long-term mortality

**DOI:** 10.1186/s13098-024-01329-5

**Published:** 2024-04-10

**Authors:** Tomás González-Vidal, Carmen Lambert, Ana Victoria García, Elsa Villa-Fernández, Pedro Pujante, Jessica Ares-Blanco, Edelmiro Menéndez Torre, Elías Delgado-Álvarez

**Affiliations:** 1 Department of Endocrinology and Nutrition, Hospital Universitario Central de Asturias/University of Oviedo, Oviedo, Spain; 2 Instituto de Investigación Sanitaria del Principado de Asturias (ISPA), Oviedo, Spain; 3 Department of Medicine, University of Oviedo, Oviedo, Spain; 4 Centre for Biomedical Network Research on Rare Diseases (CIBERER), Instituto de Salud Carlos III, Madrid, Spain

**Keywords:** Hypoglycemia, Hyperosmolar hyperglycemic state, Diabetic ketoacidosis, Mortality

## Abstract

**Background:**

Previous research has indicated that hypoglycemia during hospitalization is a predictor of unfavorable outcomes in patients with diabetes. However, no studies have examined the long-term impact of hypoglycemia in adults admitted for hyperglycemic crises. The study was aimed to investigate the long-term implications of hypoglycemia during hyperosmolar hyperglycemic crises, particularly in terms of all-cause mortality.

**Methods:**

This retrospective cohort study included 170 patients (82 men [48.2%], median age 72 years) admitted to a university hospital for hyperosmolar hyperglycemic crises, including pure hyperosmolar hyperglycemic states and hyperosmolar diabetic ketoacidoses. We separately investigated the prognostic significance of hypoglycemia on mortality during the initial intravenous insulin therapy phase and during the later subcutaneous insulin therapy phase, both during hospitalization and in the long term (median follow-up, 652 days; range 2–3460 days).

**Results:**

Both hypoglycemia during the initial intravenous insulin therapy phase (observed in 26.5% of patients) and hypoglycemia during the later subcutaneous insulin therapy phase (observed in 52.7% of patients) were associated with long-term mortality. After adjusting for potential confounders, hypoglycemia during the initial intravenous insulin therapy phase remained associated with mortality (hazard ratio 2.10, 95% CI 1.27–3.46, p = 0.004).

**Conclusions:**

Hypoglycemia during hyperosmolar hyperglycemic crises is a marker of long-term mortality, especially when it occurs during the initial intravenous insulin therapy phase.

**Supplementary Information:**

The online version contains supplementary material available at 10.1186/s13098-024-01329-5.

## Introduction

Hyperglycemic crises, such as a hyperosmolar hyperglycemic state (HHS) and diabetic ketoacidosis (DKA), are severe acute complications of diabetes mellitus [1]. HHS, which is typical but not exclusive of patients with type 2 diabetes mellitus (T2DM), is characterized by marked hyperglycemia, hyperosmolarity, and severe dehydration [2]. DKA, typical but not exclusive of patients with type 1 diabetes mellitus (T1DM) or insulin-deficient diabetes [3], is characterized by hyperglycemia, metabolic acidosis with elevated anion gap due to increased ketone body concentrations, and variable degrees of osmolality and dehydration [1]. Both entities share common symptoms, such as weight loss, polydipsia, and polyuria [4], the latter being a major cause of dehydration. In addition, infectious symptoms can be common to both conditions, given that infections often act as precipitants of these crises [5]. However, there are some differences in the symptom presentation of  HHS and DKA. The course of HHS is generally slow and insidious, whereas that of DKA is typically abrupt [6]. This is because the accumulation of ketones that occurs in DKA can produce acute symptoms characteristic of DKA: acetone breath, Kussmaul breathing, vomiting, and abdominal pain [4]. This acute symptomatology can lead patients with DKA to seek medical attention quickly, so the polyuria of DKA usually has a short evolution time and produces only a state of mild hypovolemia. However, if the DKA is not treated in time, hyperglycemia and dehydration can progress, producing a hyperosmolar state similar to that of HHS, which can contribute to altered mental status [7]. Therefore, cases can overlap between HHS and DKA [5, 8–10].

Both HHS and hyperosmolar DKA, which can be collectively referred to as hyperosmolar hyperglycemic crises (HHC), have a high mortality rate [8], including in the long term [11, 12]. HHC must be treated intensively with fluids and insulin [1]. In the initial phase, intravenous insulin therapy is recommended, with a transition to subcutaneous insulin therapy when the hyperglycemia is corrected and the patient is able to tolerate oral feeding [1]. Intensive insulin therapy carries a risk of hypoglycemia during treatment of hyperglycemic crises. In fact, hypoglycemia, along with hypokalemia, is the most common complication of hyperglycemic crises [8]. Several studies have shown that the incidence of hypoglycemia during hyperglycemic crises depends on the dosage and amount of insulin received [13–20]. In addition, 2 studies performed in patients admitted for DKA demonstrated a higher incidence of hypoglycemia in patients with low body weight [18], fever [21], hepatic disease [21], and impaired renal function [18, 21]. Thus, few studies have analyzed risk factors other than insulin therapy for the occurrence of hypoglycemia during the treatment of hyperglycemic crises.

Hypoglycemia alone can lead to severe symptoms, such as seizures, coma, or even death [22]. In addition, particularly during HHC, too rapid correction of hyperglycemia, and thus osmolality, could lead to cerebral edema, a rare but potentially fatal complication in adults [23]. For these reasons, it is essential to avoid hypoglycemia during HHC, and it is recommended that glucose should not be lowered below 200–300 mg/dL during the first few hours of treatment [1], which takes place during the initial intravenous insulin therapy phase (IIITP).

Previous studies in patients hospitalized for any reason have shown that hypoglycemia is a marker of poor short- and long-term prognosis [24–26]. However, only 1 study has analyzed the prognostic value of hypoglycemia specifically during the management of hyperglycemic crises in adult patients, showing that hypoglycemia of less than 40 mg/dL within 48 h of admission is associated with increased mortality during hospitalization [8]. To our knowledge, no studies in adults have analyzed the long-term prognostic value of hypoglycemia during hyperglycemic crises. The aim of the present study was to analyze, in patients admitted for HHC, the risk factors for the occurrence of hypoglycemia and the mid- and long-term prognostic value of hypoglycemia during the treatment of HHC, both in the IIITP and in the later subcutaneous insulin therapy phase (LSITP). To our knowledge, this is the first study of these characteristics.

## Methods

### Study design and setting

This was a retrospective cohort study conducted on adult patients who were hospitalized for HHC at a university hospital in Spain from June 2014 to October 2023. All the patients included fulfilled HHC criteria on admission, as adapted from previous criteria [1, 2, 8]: blood glucose > 1000 mg/dL, or blood glucose > 500 mg/dL and plasma osmolality ≥ 315 mOsm/kg. Among the patients with HHC, those with metabolic acidosis (pH ≤ 7.30 and bicarbonate ≤ 18 mEq/L) with positive ketonemia (point-of-care β-hydroxybutyrate levels ≥ 3 mmol/L) or, failing that (n = 4), positive ketonuria (≥ 3 + intensity), were considered to have hyperosmolar DKA. The remaining patients with HHC were considered to have pure HHS [1, 2, 8, 27]. Given that all the patients were admitted for symptomatic hyperglycemia ≥ 200 mg/dL, all patients had a diagnosis of diabetes on admission [28]. Patients were classified as having T1DM, T2DM, or diabetes secondary to pancreatic disease according to the American Diabetes Association (ADA) criteria [28]. All patients admitted with hyperosmolar DKA were tested for pancreatic autoimmunity and/or C-peptide to differentiate T1DM from T2DM [28].

We reviewed hospital admissions between June 2014 and December 2015 coded as 249.1, 250.1, 249.2, and 250.2; admissions between January 2016 and October 2023 coded as E08.1, E09.1, E10.1, E11.1, E13.1, E08.0, E09.0, E11.0, and E13.0; and all Endocrinology admissions between June 2014 and October 2023. The search identified 174 adults who met the above-mentioned criteria for HHC. For patients admitted more than once for HHC, the first admission meeting HHC criteria was selected. Four patients were excluded from the study because they received insulin therapy for less than 24 h due to death during the first hours after admission, thus having a very short exposure time to insulin therapy for hypoglycemia onset. A total of 170 patients (82 [48.2%] men, median age 72 years, range 19–98 years) met the criteria and were included in the study.

Consensus recommendations for the treatment of HHC were followed for all patients: first an IIITP, then transition to a LSITP when the clinical situation allowed [1]. Seven patients died during the IIITP, without receiving subcutaneous insulin. The hospital's standard protocol for monitoring glycemic control in patients admitted for HHC involves taking 5–10 capillary blood glucose measurements per day.

### Main determinations

#### Outcomes

##### In-hospital hypoglycemia

A patient was considered to have hypoglycemia when he/she had at least 1 capillary blood glucose determination of ≤ 69 mg/dL during hospitalization. According to the ADA classification [22], level 1 hypoglycemia was defined as a blood glucose level between 54 and 69 mg/dL, and level 2 hypoglycemia was defined as a blood glucose level < 54 mg/dL. The total days of hospitalization with at least 1 determination in the hypoglycemia range were recorded; based on these data, the percentage of hospitalization days with hypoglycemia was calculated, using the total duration of hospitalization as the denominator.

##### In-hospital hypoglycemia during the IIITP

A patient was considered to have had hypoglycemia during the IIITP if he/she had at least 1 capillary blood glucose determination of ≤ 69 mg/dL from the time of admission to the time of discontinuation of intravenous insulin therapy. For patients who experienced hypoglycemia during the IIITP, the lowest capillary glucose value recorded during that phase was registered.

##### In-hospital hypoglycemia during the LSITP

A patient was considered to have had hypoglycemia during the LSITP if he/she had at least 1 capillary blood glucose determination of ≤ 69 mg/dL from the time of discontinuation of intravenous insulin therapy (i.e., the beginning of the subcutaneous insulin therapy) until discharge from the hospital. For patients who experienced hypoglycemia during the LSITP, the lowest capillary glucose value recorded during that phase was registered.

##### Length of hospital stay

It was calculated as the number of days elapsed from the date of admission to the date of discharge.

##### Metabolic control after hospital discharge

Follow-up glycated hemoglobin (HbA1c) was the first available determination after hospital discharge, provided that at least 2 months and no more than 12 months had elapsed after discharge. A patient was considered to have good glycemic control at discharge if he/she had a follow-up HbA1c < 7% [22].

##### In-hospital and long-term mortality

Mortality during hospitalization was recorded. Long-term all-cause mortality was also registered using the electronic clinical record, which registers all patient contacts with the public health system (including hospital admissions and consultations, primary care visits, and mortality). Follow-up was extended until January 2024. Survival was calculated from the day of hospital admission until death or until the last reliable contact with the health system to account for possible misclassification due to change of address. Median follow-up was 652 days (range 2–3460 days).

### Covariates

The following variables were registered from each patient: age, sex, serum glucose on admission, serum osmolality on admission (if not determined, it was calculated by using the following formula: Sodium [in mEq/L] × 2 + Glucose [in mg/dL]/18 + Urea [in mg/dL]/6), serum potassium on admission (a patient was considered to have hypokalemia if serum potassium levels were ≤ 3.5 mmol/L, and hyperkalemia if potassium levels were ≥ 5.5 mmol/L), glomerular filtration rate on admission (calculated from serum creatinine levels applying the CKD-EPI formula) [29], body mass index (BMI), pre-existing diagnosis of diabetes prior to admission (as per the ADA criteria) [28], previous ambulatory treatment with insulin, baseline HbA1c value (obtained during hospitalization or, failing that, the most recent value available in the previous year), Charlson Comorbidity Index (CCI, an estimator that includes age, baseline glomerular filtration rate, previous history of diabetes, myocardial infarction, heart failure, peripheral vascular disease, cerebrovascular accident, hemiplegia, dementia, chronic obstructive pulmonary disease, connective tissue disease, acquired immunodeficiency syndrome, peptic ulcer disease, liver disease, solid tumor, leukemia, and lymphoma) [30], admission to an intensive care unit (ICU), presence of a probable infection (a patient was considered to have a probable infection if he/she had a fever or received antibiotic treatment during hospitalization), international units (IU) of intravenous insulin received in the first 24 h of admission, duration (hours) of the  IIITP, IU of subcutaneous insulin received in the first 24 h of the LSITP, and other treatments received during hospitalization (oral or intravenous corticosteroids, sulfonylureas, meglitinides, metformin, dipeptidyl peptidase-4 inhibitors, sodium-glucose cotransporter-2 inhibitors, glucagon-like peptide-1 agonists, and thiazolidinediones).

### Statistical analyses

We employed the chi-squared test to compare proportions, the Mann–Whitney test to compare numerical data between independent groups, and Spearman’s rank test to evaluate correlation. We employed Kaplan–Meier curves for the assessment of long-term mortality rates according to hypoglycemia during admission, and the log-rank test for between-group comparisons. Cox regression (proportional hazards regression) was used for multivariate analysis of survival during follow-up. Logistic regression was used for multivariate analyses of factors associated with mortality risk at a given time point. In those models, age was entered in years, CCI in points, and IIITP duration in hours. Age was not factored into the models including the CCI because age is a component of this index. The remaining variables were entered into the models as binary (yes/no), including sex (reference category, female), infection (reference category, no), HHC variant (reference category, hyperosmolar DKA), and form of hypoglycemia during hospitalization (reference category, no). Covariates were forced to enter the equation in all multivariate models. The proportional hazards assumption was checked using statistical tests and graphical diagnostics based on the Schoenfeld residuals. All tests were 2-tailed. P-values lower than 0.05 were considered statistically significant.

## Results

### Characteristics of the patients admitted for HHC

Of the 170 patients admitted for HHC, 125 (73.5%) had T2DM, 42 (24.7%) had T1DM, and 3 (1.8%) had diabetes secondary to pancreatic disease. Table 1 compares the characteristics of the patients with pure HHS with the patients with hyperosmolar DKA. Compared with the patients admitted with hyperosmolar DKA, the patients with pure HHS were older, had a  higher frequency of diabetes unknown prior to admission, higher T2DM frequency, higher BMI, lower hyperkalemia frequency, higher CCI (particularly, lower baseline glomerular filtration rate, and a higher frequency of heart failure, cerebrovascular accident, and cancer history), and, as expected from the diagnostic criteria, higher pH, higher bicarbonate levels, and lower β-hydroxybutyrate levels (Table 1).

**Table 1:** Comparison of patients with pure hyperglycemic hyperosmolar state (HHS) and hyperosmolar diabetic ketoacidosis (DKA)

Characteristic	Pure HHS (n = 116)	Hyperosmolar DKA (n = 54)	P-value
Demographics			
Age (years)	76 (64–83)	57 (42–73)	< 0.001
Sex (male)	54 (46.5)	28 (51.8)	0.520
Metabolic characteristics			
Known diagnosis of any form of diabetes before admission (yes)	76 (65.5)	46 (85.1)	0.008
Diagnosis of type 2 diabetes (yes)	105 (90.5)	20 (37.0)	< 0.001
Body mass index (kg/m^2^)^a^	26.9 (23.8–31.1)	23.2 (20.2–26.8)	< 0.001
HbA1c during or before admission (%)^b^	10.5 (9.2–12.6)	11.0 (9.2–13.6)	0.223
Test on admission			
Serum glucose (mg/dL)	802 (669–1002)	906 (726–1016)	0.056
Serum osmolality (mOsm/kg)	328 (320–364)	330 (320–345)	0.583
Estimated glomerular filtration rate (mL/min/1.7 m^2^)^c^	28 (20–41)	31 (21–40)	0.509
Hypokalemia (yes)^d^	8 (7.5)	4 (8.3)	0.866
Hyperkalemia (yes)^d^	25 (23.5)	21 (43.7)	0.011
pH	7.38 (7.32–7.41)	7.02 (6.92–7.16)	< 0.001
Serum bicarbonate (mEq/L)	23.4 (19.5–26.6)	8.7 (7.2–12.8)	< 0.001
Serum β-hydroxybutyrate (mmol/L)	0.5 (0.2–1.7)	6.2 (5.5–7.2)	< 0.001
Comorbidities			
Charlson comorbidity index (score points)	6 (4–7)	2 (1–5)	< 0.001
Baseline estimated glomerular filtration rate (mL/min/1.7 m^2^)^e^	54 (34–73)	78 (59–105)	< 0.001
Personal history of heart failure (yes)	22 (18.9)	2 (3.7)	0.008
Personal history of myocardial infarction (yes)	9 (7.7)	7 (12.9)	0.279
Personal history of cerebrovascular accident (yes)^f^	32 (27.5)	3 (5.5)	< 0.001
Personal history of cancer (yes)	18 (15.5)	2 (3.7)	0.026
Probable infection during admission (yes)^g^	61 (52.5)	29 (53.7)	0.892
Outcomes			
Length of hospital stay (days)	8 (5–13)	7 (5–11)	0.341
HbA1c after admission (%)^h^	6.5 (5.5–9.0)	8.5 (6.7–9.5)	0.012
Mortality during hospitalization (yes)	8 (6.8)	3 (5.5)	0.741
1-year mortality (yes)^i^	43 (40.5)	7 (15.9)	0.004
2-year mortality (yes)^j^	53 (53.0)	8 (19.5)	< 0.001

Data are absolute numbers and percentages (within parentheses) or medians and interquartile ranges (within parentheses)

^a^Calculated only if the height was available in any time, and if the body weight was available during hospitalization. Data available for 120 patients

^b^Preferably, HbA1c value during hospitalization was used. If not available, the most recent value available in the previous year was used. Data available for 163 patients

^c^Glomerular filtration rate was estimated by CKD-EPI equation, using the creatinine value on admission

^d^Data available for 154 patients, as 16 patients had serum potassium repeatedly indeterminable due to hemolysis in the admission tests

^e^Baseline glomerular filtration rate was estimated by CKD-EPI equation, using the most recent creatinine value before admission. Data available for 158 patients

^f^Includes strokes and transient ischemic attacks

^g^Probable infection was defined by the presence of fever or use of antibiotic treatment during hospitalization

^h^Follow-up HbA1c was the first available measurement after hospital discharge, provided that at a minimum of 2 months and a maximum of 12 months had elapsed after discharge. Data available for 68 patients

^i^Includes patients that reached 1 year of follow-up or died before 1 year (n = 150)

^j^Includes patients that reached 2 years of follow-up or died before 2 years (n = 141)

### Prevalence of hypoglycemia during hospitalization for HHC and associated risk factors

A total of 106 (62.4%) patients experienced hypoglycemia during hospitalization; 53 (31.2%) presented only level 1 hypoglycemia, and 53 (31.2%) patients presented at least 1 episode of level 2 hypoglycemia. Forty-five (26.5%) patients developed hypoglycemia during the IIITP. Of the 163 patients who received insulin during the LSITP, 86 (52.7%) developed hypoglycemia during this phase.

Table 2 shows a comparison of patients with and without hypoglycemia at any time during hospitalization, during the IIITP, and during the LSITP. Patients who had hypoglycemia during the IIITP had lower BMI, lower frequency of hyperkalemia, higher intravenous insulin doses in the first 24 h of admission, and longer IIITP duration. Patients who had hypoglycemia during the LSITP were older, had higher CCI, higher frequency of ambulatory treatment with insulin prior to admission, lower serum osmolality on admission, higher frequency of infection during admission, and higher subcutaneous insulin doses in the first 24 h of the LSITP. A history of myocardial infarction was more frequent in patients who had hypoglycemia during any time of hospitalization. There was no significant association between the occurrence of hypoglycemia and any of the other variables listed in Table 2: sex, known diagnosis of diabetes before admission, type of diabetes, other tests on admission (HbA1c, serum glucose, and glomerular filtration rate), history of heart failure, cerebrovascular accident or cancer, type of hyperglycemic crisis (pure HHS or hyperosmolar DKA), ICU admission, use of non-insulin antidiabetic agents, or use of systemic corticoids (which were used in 36 patients, primarily as supportive therapy for infections [n = 11], supportive therapy for cancer [n = 10], and immunosuppressive therapy for solid organ transplantation [n = 5]).

**Table 2:** Characteristics of patients with hypoglycemia during hospitalization for hyperosmolar hyperglycemic crises

	Hypoglycemia during any time of hospitalization			Hypoglycemia during the initial intravenous insulin therapy phase			Hypoglycemia during the later subcutaneous insulin therapy phase		
Characteristic	No (n = 64)	Yes (n = 106)	P value	No (n = 125)	Yes (n = 45)	P value	No (n = 77)	Yes (n = 86)	P value
Demographics									
Age (years)	66 (57–79)	74 (58–83)	0.140	71 (59–81)	74 (52–84)	0.712	65 (56–79)	74 (59–83)	0.013
Sex (male)	31 (48.4)	51 (48.1)	0.967	59 (47.2)	23 (51.1)	0.653	41 (53.2)	39 (45.3)	0.314
Metabolic characteristics									
Known diagnosis of any form of diabetes before admission (yes)	42 (65.6)	80 (75.4)	0.167	87 (69.6)	35 (77.7)	0.296	52 (67.5)	65 (75.5)	0.254
Ambulatory treatment with insulin before admission (yes)	20 (31.2)	55 (51.8)	0.009	51 (40.8)	24 (53.3)	0.147	25 (32.4)	46 (53.4)	0.007
Diagnosis of type 2 diabetes (yes)	51 (79.6)	74 (69.8)	0.157	95 (76.0)	30 (66.7)	0.224	59 (76.6)	60 (69.7)	0.325
Body mass index (kg/m^2^)^a^	27.3 (24.1–30.8)	25.0 (22.0–28.7)	0.009	26.8 (23.5–31.1)	23.1 (20.4–25.9)	< 0.001	26.7 (23.3–29.9)	25.1 (22.5–30.1)	0.284
HbA1c during or before admission (%)^b^	10.8 (9.3–13.2)	10.6 (9.2–12.6)	0.734	10.5 (9.2–12.8)	11.0 (9.2–12.8)	0.794	11.2 (9.8–13.3)	10.5 (9.2–12.4)	0.228
Biochemical tests on admission									
Serum glucose (mg/dL)	809 (679–992)	840 (684–1017)	0.930	814 (679–994)	866 (686–1044)	0.670	815 (686–993)	824 (671–1006)	0.756
Serum osmolality (mOsm/kg)	334 (321–354)	326 (319–353)	0.226	328 (319–348)	335 (322–375)	0.055	331 (322–354)	325 (318–344)	0.032
Estimated glomerular filtration rate (mL/min/1.7 m^2^)^c^	29 (21–44)	28 (20–38)	0.684	31 (22–42)	27 (17–37)	0.108	28 (21–45)	31 (22–38)	0.983
Hypokalemia (yes)^d^	5 (8.6)	7 (7.2)	0.766	8 (7.2)	4 (9.3)	0.663	7 (9.0)	5 (5.8)	0.440
Hyperkalemia (yes)^d^	16 (27.5)	30 (31.2)	0.630	39 (35.1)	7 (16.2)	0.022	19 (24.6)	26 (30.2)	0.383
Comorbidities									
Charlson Comorbidity Index (score points)	4 (2–6)	5 (3–7)	0.012	5 (3–7)	5 (2–7)	0.880	4 (2–6)	5 (4–7)	0.004
Baseline estimated glomerular filtration rate (mL/min/1.7 m^2^)^e^	60 (42–83)	63 (38–82)	0.839	61 (40–82)	66 (38–84)	0.796	63 (43–85)	61 (38–78)	0.464
Personal history of heart failure (yes)	6 (9.4)	18 (17.0)	0.168	16 (12.8)	8 (17.8)	0.411	9 (11.7)	14 (16.3)	0.401
Personal history of myocardial infarction (yes)	1 (1.6)	15 (14.2)	0.006	11 (8.8)	5 (11.1)	0.649	4 (5.2)	12 (14.0)	0.061
Personal history of cerebrovascular accident (yes)^f^	10 (15.6)	25 (23.6)	0.214	25 (20.0)	10 (22.2)	0.752	12 (15.6)	22 (25.6)	0.117
Personal history of cancer (yes)	6 (9.4)	14 (13.2)	0.452	16 (12.8)	4 (8.9)	0.485	8 (10.4)	12 (14.0)	0.489
Probable infection during admission (yes)^g^	27 (42.1)	63 (59.4)	0.029	64 (51.2)	26 (57.7)	0.448	32 (41.5)	53 (61.6)	0.010
Characteristics of admission									
Hyperosmolar ketoacidosis (yes)^h^	17 (26.6)	37 (28.3)	0.258	38 (30.4)	16 (35.5)	0.524	23 (29.8)	30 (34.8)	0.495
ICU admission (yes)	12 (18.7)	18 (16.9)	0.769	22 (17.6)	8 (17.7)	0.979	15 (19.4)	14 (16.2)	0.594
Therapy during hospitalization									
Intravenous insulin dose in the first 24 h of hospitalization (IU/kg)^i^	1.0 (0.6–1.5)	1.4 (1.0–1.8)	0.030	1.2 (0.7–1.6)	1.5 (1.1–2.1)	0.017	NA	NA	NA
Subcutaneous insulin dose in the first 24 h of subcutaneous therapy (IU/kg)^j^	0.6 (0.5–0.8)	0.7 (0.5–1.1)	0.263	NA	NA	NA	0.6 (0.5–0.8)	0.7 (0.5–1.1)	0.008
Initial intravenous insulin therapy phase duration (hours)	44 (24–71)	50 (39–91)	0.082	46 (24–71)	69 (44–122)	< 0.001	NA	NA	NA
Secretagogues (yes)^k^	1 (1.9)	2 (2.7)	0.790	NA	NA	NA	1 (1.6)	2 (2.3)	0.569
Antidiabetic agents that are unlikely to cause hypoglycemia (yes)^l^	24 (47.0)	29 (39.1)	0.382	NA	NA	NA	27 (45.7)	26 (30.2)	0.790
Systemic corticosteroids (yes)^m^	14 (21.8)	22 (20.7)	0.862	29 (23.2)	7 (15.5)	0.282	18 (23.3)	15 (17.4)	0.347
Outcomes									
Length of hospital stay (days)	7 (5–11)	9 (6–13)	0.022	8 (5–12)	9 (6–13)	0.220	8 (5–12)	9 (6–14)	0.036
Mortality during hospitalization (yes)	4 (3.1)	7 (6.6)	0.928	4 (3.2)	7 (15.5)	0.004	2 (2.5)	2 (2.3)	0.911
1-year mortality (yes)^n^	15 (27.2)	35 (36.8)	0.231	31 (28.1)	19 (47.5)	0.026	18 (27.2)	25 (32.4)	0.499
2-year mortality (yes)^o^	19 (37.3)	42 (46.7)	0.278	38 (36.9)	23 (60.5)	0.012	23 (37.1)	31 (43.1)	0.483

Data are absolute numbers and percentages (within parentheses) or medians and interquartile ranges (within parentheses)

^a^Calculated only if the height was available in any time, and if the body weight was available during hospitalization. Data available for 120 patients

^b^Preferably, HbA1c value during hospitalization was used. If not available, the most recent value available in the previous year was used. Data available for 163 patients

^c^Glomerular filtration rate was estimated by CKD-EPI equation, using the creatinine value on admission

^d^Data available for 154 patients, as 16 patients had serum potassium repeatedly indeterminable due to hemolysis in the admission tests

^e^Baseline glomerular filtration rate was estimated by CKD-EPI equation, using the most recent creatinine value before admission. Data available for 158 patients

^f^Includes strokes and transient ischemic attacks

^g^Probable infection was defined by the presence of fever or use of antibiotic treatment during hospitalization

^h^Hyperosmolar ketoacidosis (as opposed to pure hyperosmolar hyperglycemic state) was defined by metabolic acidosis (pH ≤ 7.30 and bicarbonate ≤ 18 mEq/L) with positive ketonemia (point-of-care β-hydroxybutyrate ≥ 3 mmol/L or, failing that, ≥ 3 + ketonuria intensity)

^i^Dose of intravenous insulin received in the first 24 h of hospitalization. Data available for 126 patients

^j^Dose of subcutaneous insulin received in the first 24 h of the later subcutaneous insulin therapy phase. Data available for 127 patients

^k^Includes sulfonylureas and meglitinides

^l^Includes metformin, dipeptidyl peptidase-4 inhibitors, glucagon-like peptide-1 agonists, sodium-glucose cotransporter-2 inhibitors, and thiazolidinediones

^m^Patients who received at least one dose of any type of oral or intravenous glucocorticoid

^n^Includes patients that reached 1 year of follow-up or died before 1 year (n = 150)

^o^Includes patients that reached 2 years of follow-up or died before 2 years (n = 141)

*ICU* intensive care unit, *NA* not applicable

### Longitudinal studies in relation to hypoglycemia during hospitalization for HHC

#### Length of hospital stay

The patients who had hypoglycemia during hospitalization had longer hospital stays than those who did not (Table 2). However, there was no significant correlation between the days of hospitalization and the proportion of days of hospitalization with at least 1 episode of hypoglycemia (Rho = 0.036; p = 0.646).

#### Glycemic control after hospital discharge

Follow-up HbA1c was available for 68 patients discharged from the hospital. Mean follow-up HbA1c was lower in patients with T2DM (n = 45) than in patients with T1DM (n = 23) (6.9% versus 9.2%, respectively; p < 0.001). Thirty-three (48.5%) patients had good glycemic control at discharge (i.e., follow-up HbA1c < 7%).

Follow-up HbA1c was higher in patients who had level 2 hypoglycemia during hospitalization (n = 16; mean HbA1c, 8.9%) than in those who did not (n = 52; mean HbA1c, 7.4%; p = 0.007). When selecting patients with T2DM, mean follow-up HbA1c was also higher in patients who had level 2 hypoglycemia (n = 7; mean HbA1c 8.4%) than in those who did not (n = 38; mean HbA1c 6.7%; p = 0.040). When selecting patients with available follow-up HbA1c who experienced hypoglycemia during hospitalization (n = 35), the lowest capillary glucose value recorded during hospitalization was lower in patients who had poor glycemic control (HbA1c ≥ 7%) after discharge (n = 21; mean 47 mg/dL) than in those who had good glycemic control after discharge (n = 14; mean 58 mg/dL; p = 0.004). There was a directly proportional correlation between the percentage of hospitalization days with hypoglycemia and the follow-up HbA1c level (Rho = 0.268, p = 0.027).

#### All-cause mortality

A total of 90 (52.9%) patients died during the whole follow-up: 50 died during the first year of follow-up (11 died during hospitalization and 39 died after discharge), 11 died between the first and second year of follow-up, and 29 died after the second year of follow-up. Patients with pure HHS had higher mortality than patients with hyperosmolar DKA (Table 1). Mortality during hospitalization was higher in patients who had hypoglycemia during the IIITP than in those who did not (Table 2). When selecting patients who experienced hypoglycemia during the IIITP (n = 45), the lowest capillary glucose value recorded during the IIITP was lower in patients who died during hospitalization (mean, 43 mg/dL) than in those who did not (mean, 54 mg/dL; p = 0.022).

**Fig. 1:**
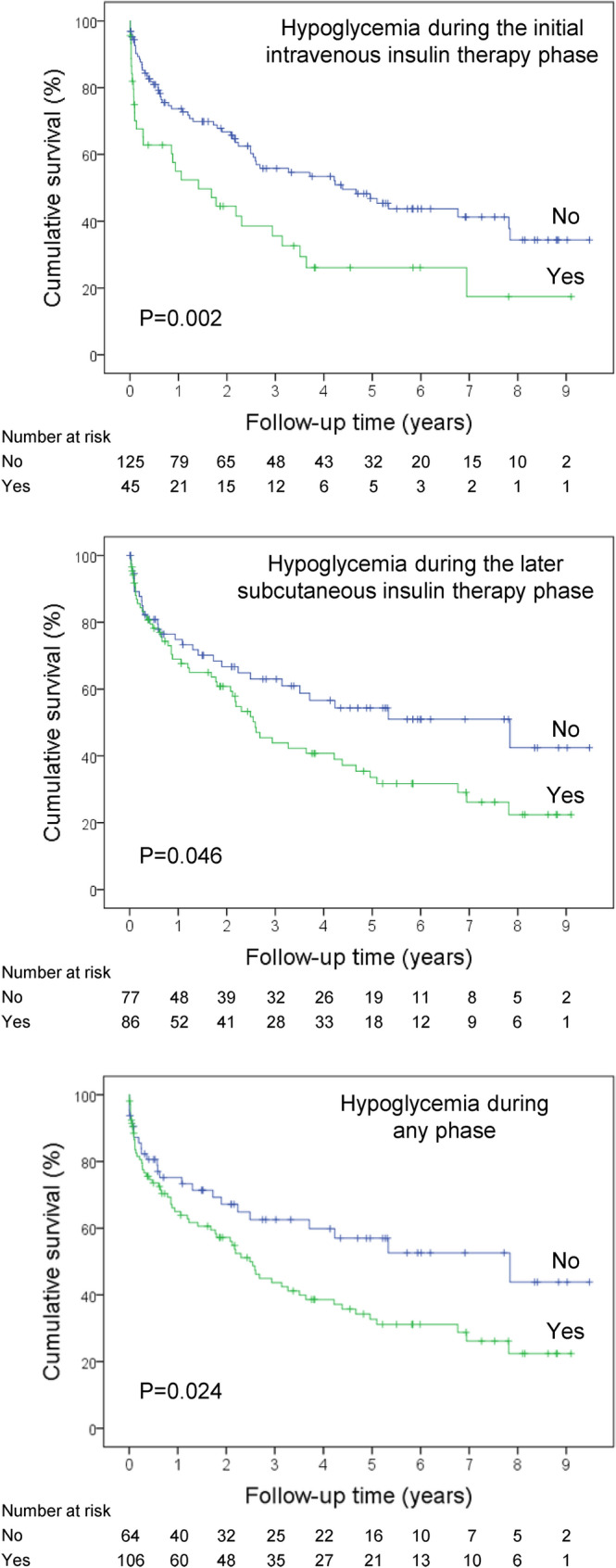
Probability of survival (Kaplan–Meier curves) in patients stratified according to the various forms of hypoglycemia during hospitalization for hyperosmolar hyperglycemic crises. Vertical ticks represent censored data. P-values were calculated with the log-rank test

Mortality during the follow-up period was higher among those who experienced hypoglycemia during the IIITP compared to those who did not (Fig. 1). Similarly, in the univariate survival analysis, mortality during follow-up was higher in patients who developed hypoglycemia during the LSITP (Fig. 1).

On Cox regression analysis (proportional risk model), the association between hypoglycemia during the IIITP and mortality risk persisted after adjusting for covariates, including age, sex, CCI, diagnosis of pure HHS (versus hyperosmolar DKA), infection during hospitalization, and IIITP duration (Table 3). In these models, older age, higher CCI, and a diagnosis of pure HHS were significant risk factors for long-term mortality (data not shown; Additional file 1). However, after adjusting for the aforementioned covariates, the association between hypoglycemia during the LSITP and mortality risk was no longer present (Table 3).

**Table 3:** Multivariate analyses of factors associated with mortality (Cox regression)

Hypoglycemia during hospitalization	Crude analyses		Adjusted for age and sex		Adjusted for Charlson Comorbidity Index		Multiadjusted^a^	
	HR (95% CI)	P-value	HR (95% CI)	P-value	HR (95% CI)	P-value	HR (95% CI)	P-value
Hypoglycemia during the initial intravenous insulin therapy phase	1.94 (1.24–3.01)	0.003	1.83 (1.17–2.88)	0.008	2.61 (1.65–4.14)	< 0.001	2.10 (1.27–3.46)	0.004
Hypoglycemia during the later subcutaneous insulin therapy phase	1.56 (1.00–2.44)	0.048	1.11 (0.70–1.74)	0.649	1.16 (0.74–1.81)	0.517	1.20 (0.75–1.92)	0.428
Hypoglycemia during any phase of hospitalization	1.69 (1.06–2.68)	0.025	1.34 (0.84–2.15)	0.217	1.36 (0.85–2.16)	0.189	1.40 (0.87–2.26)	0.157

^a^Adjusted for sex, Charlson Comorbidity Index, pure hyperglycemic hyperosmolar state (versus diabetic ketoacidosis), probable infection during admission, and initial intravenous insulin therapy phase duration. Age was not added to models including the Charlson Comorbidity Index because age is a component of the index. The models included 170 cases, except models with hypoglycemia during the later subcutaneous insulin therapy phase, which included 163 cases

*HR* hazard ratio, *CI* confidence interval

Among the 150 evaluable patients, the 1-year mortality was higher in patients with hypoglycemia during the IIITP than in patients without it (relative risk 1.70, 95% CI 1.07–2.69, p = 0.026; Table 2). Likewise, among the 141 evaluable patients, the 2-year mortality was higher in the patients with hypoglycemia during the IIITP than in those without it (relative risk 1.64, 95% CI 1.14–2.35, P = 0.012; Table 2). On multivariate analysis (logistic regression), hypoglycemia during the IIITP was associated with both 1-year and 2-year mortality after adjusting for covariates (data not shown; Additional file 1).

## Discussion

To date, only 1 study had examined the prognostic value of hypoglycemia in adults admitted for hyperglycemic  crises, finding that hypoglycemia of less than 40 mg/dL during the first 48 h of treatment is associated with higher in-hospital mortality [8]. A recent study of the pediatric population in Ethiopia showed that hypoglycemia during DKA is a predictor of long-term mortality in children younger than 15 years of age [31]. Our study confirmed that early hypoglycemia during HHC is associated with higher in-hospital mortality and, to our knowledge, was the first to analyze the prognostic significance of hypoglycemia during HHC on long-term mortality. Specifically, hypoglycemia during the early IIITP for HHC was independently associated with long-term mortality. In addition, the present study also analyzed the risk factors associated with the occurrence of hypoglycemia during HHC and the effect of hypoglycemia during HHC on glycemic control after hospital discharge.

Our results showed that hypoglycemia is common during hospitalizations for HHC, occurring in more than half of the patients, particularly during the LSITP. The frequency of hypoglycemia during the IIITP (mean duration, 67 h) in our sample (present in about a quarter of patients) was proportionally similar to that observed in the first 48 h in previous studies (12–16%) [8]. Consistent with previous studies in patients hospitalized for hyperglycemic  crises, hypoglycemia was more frequent in patients with low BMI [18], in patients with probable concomitant infection [21], and in those receiving higher doses of insulin [17]. Additional risk factors found in our study for hypoglycemia during HHC were older age, the presence of a greater number of comorbidities (higher CCI), and outpatient treatment with insulin prior to admission; these had not been reported in patients admitted for hyperosmolar crises, but had been documented in patients with diabetes who were admitted for other reasons [24]. Routine blood tests on admission were not associated with hypoglycemia, except for serum potassium concentrations: patients who had hypoglycemia during the IIITP had a lower frequency of hyperkalemia. The reason for this association is unknown, since patients with hyperkalemia received similar doses of intravenous insulin and had a similar glomerular filtration rate as patients with hypo- or normokalemia (data not shown; Additional file 1). Future studies are needed to confirm this association and the reason for this effect.

Our study showed that hypoglycemia during HHC is associated with long-term all-cause mortality; however, only hypoglycemia during the IIITP maintained the association with mortality risk after adjusting for confounders. The reasons why hypoglycemia during HHC therapy is associated with mortality are not entirely known. In the early acute phase (i.e., during the IIITP), abrupt changes in plasma osmolality caused by rapid correction of hyperglycemia can lead to neurological damage [23]. Although no cases of cerebral edema were described in our series, we cannot rule out the possibility that these early hypoglycemic episodes after an abrupt change in osmolality could have left some kind of neuronal imprint that contributes to increased mortality. It is well known that hypoglycemia, even when asymptomatic, can cause a vicious cycle of recurrent hypoglycemia by leading to hypoglycemia-associated autonomic failure, the clinical syndrome of deficient glucose counter-regulation and impaired awareness of hypoglycemia [32]. Previous studies have shown that hypoglycemia in individuals with T2DM [33–36] and T1DM [35] is associated with an increased risk of cardiovascular disease. Our study also showed that a history of myocardial infarction is associated with hypoglycemia during HHC, independently of the insulin dose received (data not shown; Additional file 1). The reason for this finding is unknown, but could be related to cardiovascular disorders associated with hypoglycemia [32]. Moreover, previous studies have demonstrated that hypoglycemia (particularly, severe hypoglycemia requiring external assistance) is associated with long-term mortality [35–38], including all-cause and cardiovascular mortality, but also with noncardiovascular mortality [36, 37]. Of note, the relative risk (hazard ratio) of mortality associated with hypoglycemia in these studies was between 2- and threefold [35, 36, 38], similar to that observed in the present study. Associations between hypoglycemia and mortality might not establish a causal connection [39]. Hypoglycemia could be a marker of serious underlying disease rather than a cause of death [36, 39]. However, in some studies hypoglycemia was associated with mortality after adjusting for the CCI [38], as we did in the present study. Another factor that might contribute to long-term mortality in patients with hypoglycemia is poorer glycemic control at discharge, which our study found in patients with level 2 hypoglycemia during HHC treatment. The reason why patients with level 2 hypoglycemia had higher follow-up HbA1c levels could be that the hypoglycemic treatment at discharge might have been less intense in this group of patients to prevent new episodes of hypoglycemia. Future studies are necessary to clarify the pathways by which hypoglycemia during the IIITP of HHC is associated with short- and long-term mortality.

The study has limitations that must be acknowledged, including those that are common to all retrospective studies (particularly, information bias and the possibility that confounding factors were not adequately controlled) [40]. Post-discharge glycemic control was assessed in terms of HbA1c up to 12 months after discharge, which may be too long a time to consider that HbA1c at that point was influenced by hypoglycemia during hospitalization. In terms of strengths, it should be taken into account that this is a real-life study, with a uniform therapeutic protocol and prolonged follow-up.

## Conclusions

Hypoglycemia during HHC is a marker of long-term all-cause mortality, especially when it occurs during the IIITP. The results support the recommendation not to excessively lower glucose levels during the first hours of insulin treatment [1], with particular caution in patients at high risk of hypoglycemia during the IIITP (especially those with low BMI). The results indicate that patients who experience hypoglycemia during hospitalization are a vulnerable population with high mortality rates both during and after their hospital stay. Therefore, it is crucial to emphasize preventive measures for this group of patients. More studies will be necessary to evaluate the possible neurohormonal and cardiovascular changes that patients might have undergone during hypoglycemia after treatment for HHC, as well as to evaluate other possible mechanisms that increase mortality after this form of hypoglycemia.

### Supplementary Information

**Additional file 1. Data not shown in the main text.**

## Data Availability

The datasets used and/or analyzed during the current study are available from the corresponding author on reasonable request.

## Abbreviations

ADA: American Diabetes Association
BMI: Body mass index
CCI: Charlson Comorbidity Index
CI: Confidence interval
DKA: Diabetic ketoacidosis
HbA1c: Glycated hemoglobin
HHC: Hyperosmolar hyperglycemic crises
HHS: Hyperosmolar hyperglycemic state
ICU: Intensive care unit
IIITP: Initial intravenous insulin therapy phase
IU: International units
LSITP: Later subcutaneous insulin therapy phase
T1DM: Type 1 diabetes mellitus
T2DM: Type 2 diabetes mellitus
