# Supplementary material for: Hypoglycemia during hyperosmolar hyperglycemic crises is associated with long-term mortality
Source: Diabetol Metab Syndr. 2024 Apr 10;16:83. doi: 10.1186/s13098-024-01329-5 (PMC11005231; doi:10.1186/s13098-024-01329-5)
Supplement: Supplementary file 1 — Additional file 1. Data not shown in the main text. [file 13098_2024_1329_MOESM1_ESM.pdf]

Multivariate analyses of factors associated with mortality (Cox regression)

	**Adjusted for age and sex**		**Adjusted for Charlson comorbidity index**		**Multiadjusted***	
	**HR** (95% CI)	**P-value**	**HR** (95% CI)	**P-value**	**HR** (95% CI)	**P-value**
**Hypoglycemia during the initial intravenous insulin phase** (yes)	1.83 (1.17-2.88)	0.008	2.61 (1.65-4.14)	<0.001	2.10 (1.27-3.46)	0.004
**Age** (years)	1.05 (1.03-1.07)	<0.001	NA	NA	NA	NA
**Sex** (men)	1.20 (0.76-1.87)	0.426	NA	NA	0.68 (0.44-1.05)	0.085
**Charlson Comorbidity Index** (points)	NA	NA	1.32 (1.23-1.42)	<0.001	1.31 (1.20-1.42)	<0.001
**Pure hyperglycemic hyperosmolar state** (yes)	NA	NA	NA	NA	2.18 (1.18-4.03)	0.013
**Probable infection during admission** (yes)	NA	NA	NA	NA	1.33 (0.84-2.12)	0.220
**Initial intravenous insulin therapy phase duration** (hours)	NA	NA	NA	NA	1.00 (1.00-1.01)	0.083

*Adjusted for sex, Charlson Comorbidity Index, pure hyperglycemic hyperosmolar state (versus diabetic ketoacidosis), probable infection during admission, and initial intravenous insulin therapy phase duration.

HR, hazard ratio. CI, confidence interval. NA, not applicable.

Multivariate analyses of factors associated with 1 and 2-year mortality (logistic regression)

**1-year mortality**[a]						
	**Adjusted for age and sex**		**Adjusted for Charlson comorbidity index**		**Multiadjusted***	
	**OR** (95% CI)	**P-value**	**OR** (95% CI)	**P-value**	**OR** (95% CI)	**P-value**
**Hypoglycemia during the initial intravenous insulin phase**	2.24 (1.01-4.96)	0.045	2.57 (1.16-5.68)	0.019	2.60 (1.15-5.87)	0.021
**2-year mortality**[b]						
	**Adjusted for age and sex**		**Adjusted for Charlson comorbidity index**		**Multiadjusted***	
	**OR** (95% CI)	**P-value**	**OR** (95% CI)	**P-value**	**OR** (95% CI)	**P-value**
**Hypoglycemia during the initial intravenous insulin phase**	2.77 (1.20-6.39)	0.017	3.13 (1.35-7.26)	0.008	3.34 (1.38-8.10)	0.007

*Adjusted for sex, Charlson Comorbidity Index, pure hyperglycemic hyperosmolar state (versus diabetic ketoacidosis), and probable infection during admission.

[a]Data available for 150 patients (20 patients did not die in the first year or had less than 365 days of follow-up).

[b]Data available for 141 patients (29 patients did not die in the first 2 years or had less than 730 days of follow-up).

OR, odds ratio. CI, confidence interval.

Patients with hyperkalemia received similar doses of intravenous insulin and had a similar glomerular filtration rate as patients with hypo- or normokalemia

Mean intravenous insulin dose in the first 24 hours of hospitalization in patients with hyperkalemia: 1.43 IU/kg
Mean intravenous insulin dose in the first 24 hours of hospitalization in patients with hypo- or normokalemia: 1.30 IU/kg
Mann-Whitney test: p=0.306

Mean glomerular filtration rate in patients with hyperkalemia: 30.3 mL/min/1.7 m^2
Mean glomerular filtration rate in patients with hypo- or normokalemia: 33.0 mL/min/1.7 m^2
Mann-Whitney test: p=0.981

A history of myocardial infarction is associated with hypoglycemia during HHC, independently of the insulin dose received

Mean intravenous insulin dose in the first 24 hours of hospitalization in patients with myocardial infarction: 1.47 IU/kg
Mean intravenous insulin dose in the first 24 hours of hospitalization in patients with no myocardial infarction: 1.34 IU/kg
Mann-Whitney test: p=0.371

Mean subcutaneous insulin dose in the first 24 hours of the LSITP in patients with myocardial infarction: 0.77 IU/kg
Mean subcutaneous insulin dose in the first 24 hours of the LSITP in patients with no myocardial infarction: 0.78 IU/kg
Mann-Whitney test: p=0.968
